# Design, Synthesis, and Characterization of Schiff Base Bond-Linked pH-Responsive Doxorubicin Prodrug Based on Functionalized mPEG-PCL for Targeted Cancer Therapy

**DOI:** 10.3390/polym10101127

**Published:** 2018-10-11

**Authors:** Yinglei Zhai, Xing Zhou, Zhiqiang Zhang, Lei Zhang, Dianyu Wang, Xinhui Wang, Wei Sun

**Affiliations:** 1 Department of Biomedical Engineering, School of Medical Devices, Shenyang Pharmaceutical University, Shenyang 110016, China; yingleizhai@syphu.edu.cn (Y.Z.); wdy545820@163.com (D.W.); m15141210344@163.com (X.W.); 2 Hainan Institute of Materia Medica, Haikou 570311, China; beyondyme@163.com; 3 Department of Pharmaceutics, School of Pharmacy, Shenyang Pharmaceutical University, Shenyang 110016, China; zifeiyuxq@163.com; 4 Shanghai Pharma Group (Benxi) Northern Pharmaceutical Co., Ltd., Benxi 117004, China; zl.719@163.com

**Keywords:** doxorubicin, pH-sensitive, prodrug, micelles, imine

## Abstract

The side effects of doxorubicin (DOX) extremely limit its application in the treatment of malignant tumors. Nano-sized polymeric drugs based on the acidic microenvironment of tissular- or intra- tumor have attracted ample attention because of their potential in reducing side effects. In this research, an amphiphilic diblock copolymer based on poly (ethylene glycol) (PEG) and functionalized polycaprolactone (PCL) was synthesized and utilized as the drug carrier. DOX was chemically conjugated with the polymer via acid-cleavable imine bonds to obtain a novel pH-sensitive DOX prodrug (mPEG-PCL-Imi-DOX). mPEG-PCL-Imi-DOX (24.2 wt % DOX content) formed micelles with an average diameter of 125 nm through a simple solvent evaporation method. The in vitro release profile demonstrated that DOX release of the prodrug micelles was pH-responsive and able to be accelerated with the decrease of pH. In vitro cytotoxicity assay tests revealed that the pH-sensitive DOX prodrug micelles exhibited relatively lower toxicity and similar antitumor efficacy towards MCF-7 cells compared with free DOX. Hence, the DOX prodrug micelles with imine bonds can offer a carrier with great potential for chemo-therapeutics.

## 1. Introduction

Anthracyline agent doxorubicin (DOX) is a highly effective antitumor chemotherapeutic, which is extensively applied in the treatment of various cancers [1,2,3]. DOX can devastate cancer cells and suppress tumor progression by inhibiting the synthesis of DNA and RNA. However, a series of defects have seriously limited its further clinical application, such as irreversible cardiotoxicity, rapid blood/renal clearance, and poor bioavailability [4,5,6,7]. During the past decades, a myriad of drug delivery systems (DDS), such as nanoparticles [8,9,10], hydrogels [11,12] and micelles [13,14] have been developed in order to overwhelm these obstacles. As with most potential DDSs, nano-sized polymeric drugs have been a hotspot because of their unique properties, such as passive accumulation in tumor tissues via the enhanced permeability and retention (EPR) effect [15], improved pharmacokinetics behaviors [16], reduced toxicity to normal tissues [17], and so forth.

The extensively reported acidic microenvironments (pH 4.0–7.0) of extra- and intra- tumor cells offers an impactful platform for the development of pH-responsive nanoparticles, which could fulfill the selective drug release into carcinomas cells [18]. The fabrication of nano-sized pH-sensitive DDS generally employs two strategies: physical encapsulation [19,20,21] and chemical conjugation [22,23]. Compared with covalent conjugation, one limitation of physical encapsulation is the relatively low stability, which will result in undesired drug leakage and systemic toxicity due to the dissociation of nanocarriers. Accordingly, the pH-sensitive covalent bonds including hydrazone [24], acetal [25], and imine [23] are widely utilized to construct the intracellular DDS. By comparison with other acid-labile linkages, the synthesis of imine bonds is more convenient and efficient despite the fact that the aliphatic imine linkage is scarcely employed to incorporate the hydrophobic drugs into the polymeric nanocarriers owing to its hydrolysis even under physiological conditions [26]. However, the aromatic imine bond can remain stable at physiological pH and tend to be easily hydrolyzed under a slightly acidic environment in contrast, which is attributed to the π-π conjugated effect [27,28].

As a ratified biomedical material by the United States Food and Drug Administration (FDA), polycaprolactone (PCL) has been extensively utilized in advanced DDS. Despite the excellent biocompatibility and biodegradability of PCL, the absence of pendant functional groups seriously limits its further application in the preparation of stimuli-responsive anti-cancer prodrugs. A newly developed functional caprolactone monomer [29] makes it possible to synthesize stimuli-responsive polymeric prodrugs.

In this article, an amphiphilic di-block copolymer based on poly (ethylene glycol) (PEG) and functionalized PCL was fabricated via ring-opening polymerization (ROP), hydrolysis and esterification, and applied as the nano-carrier for DOX. The polymeric prodrug with pH-sensitive imine bonds was expected to possess improved stability, relatively higher drug loading content, and enhanced tumor targetability, which was likely to reduce the premature drug leaching and the systemic toxicity. The chemical structures of DOX prodrugs were analyzed by ^1^H NMR and Fourier transform infrared (FT-IR) spectroscopy. The drug loading content was investigated employing ultraviolet–visible (UV-vis) spectroscopy. In addition, the particle size and distribution of DOX prodrug micelles were determined using dynamic light scattering (DLS) and transmission electron microscopy (TEM). In vitro drug release behavior according to different pH conditions, the internalization and cytotoxicity of DOX-conjugated micelles to MCF-7 cells were also investigated.

## 2. Experimental Section

### 2.1. Materials

4-Dimethylaminopyridine (DMAP), tin(II) 2-ethylhexanoate (Sn(Oct)_2_), dicyclohexylcarbodiimide (DCC), trifluoroacetic acid (TFA) and 4-hydroxy benzaldehyde were purchased from Aladdin (Shanghai, China). Doxorubicin hydrochloride (DOX·HCl) and polyethylene glycol monomethyl ether (MW = 5000, mPEG) were received from Meilun Biotechnology (Dalian, China) and Sigma-Aldrich (Shanghai, China), respectively. According to the previously reported literature [29], 3-(7-oxo-oxepan-4-yloxy)-propionic acid t-butyl ester (PABE) was synthesized. Prior to use, 1,4-dioxane was pre-dried with CaCl_2_ and then refluxed with Na wire and benzophenone under argon protection. Anhydrous methanol (MeOH) was obtained by refluxing with iodine and magnesium chips. All the other solvents of analytical grade were used directly without any further purification. For in vitro cell viability study and in vitro cellular uptake research, a human breast cancer cell line (MCF-7) was obtained from American Type Culture Collection (ATCC) (Beijing, China). RPMI Medium 1640 and Dulbecco’s Modified Eagle Medium (DMEM) were received from Thermo Fisher Scientific (Hampton, NH, USA). Fetal Bovine Serum was afforded by Biological Industries.

### 2.2. Synthesis of mPEG-PCL with Substituted Carboxylic Groups (mPEG-CPCL)

mPEG-CPCL was synthesized in two steps according to the established literature procedures [29].

#### 2.2.1. Synthesis of mPEG-BuPCL Block Copolymer

mPEG-BuPCL was synthesized by Sn(Oct)_2_ catalyzed ROP. Typically, a dry Schlenk flask was charged with macroinitiator mPEG5000 (714 mg, 0.14 mmol), functionalized caprolactone monomer PABE (921 mg, 3.57 mmol) and Sn(Oct)_2_ (29 mg, 0.07 mmol). After vacuumed and pumped in argon, the reaction mixture was stirred for 48 h at 130 °C. The polymerization mixture was cooled to room temperature and dissolved in DCM (10 mL). The polymer was precipitated in ice diethyl ether and isolated by filtration 3 times to obtain the off-white sticky solid of mPEG-BuPCL 1045 mg (conversion rate: 80%). ^1^H NMR (600 MHz, CDCl_3_) δ 4.15–4.11 (m, OCH_2_), 3.65–3.63 (m, CH_2_CH_2_O and OCH_2_), 3.45–3.41 (m, OCH), 3.37 (s, CH_3_O), 2.44–2.42 (t, COCH_2_), 2.38–2.34 (m, COCH_2_), 1.89–1.72 (m, CH(CH_2_)_2_), 1.44 (s, C(CH_3_)_3_).

#### 2.2.2. Synthesis of mPEG-CPCL

The amphiphilic mPEG-BuPCL (1.0 g) was dissolved in DCM. Subsequently, excessive trifluoroacetic acid (3.5 mL) was added dropwise by using a syringe at 0 °C. The hydrolysis was continued for another 4 h with vigorous stirring at 25 °C. The solvent was evaporated under reduced pressure and the viscous residue was redissolved in DCM (2 mL). Then, the solution was added slowly into cold diethyl ether and filtered to get pure mPEG-CPCL 828 mg (yield: 93%). ^1^H NMR (600 MHz, CDCl_3_) δ 4.17 (s, OCH_2_), 3.74–3.68 (d, CH_2_CH_2_O and OCH_2_), 3.57–3.53 (m, OCH), 3.44 (s, CH_3_O), 2.61–2.59 (t, COCH_2_), 2.41 (s, COCH_2_), 1.88–1.81 (d, CH(CH_2_)_2_).

### 2.3. Synthesis of Imine-Linked DOX Prodrugs Based on PEG-PCL di-Block Copolymer (mPEG-PCL-Imi-DOX)

#### 2.3.1. Synthesis of Aldehyde-Functionalized mPEG-PCL (mPEG-APCL)

To a 100 mL round-bottom flask, mPEG-CPCL (890 mg, equiv. 1.97 mmol COOH) was dissolved in anhydrous 1,4-dioxane (50 mL). The resulting solution was cooled to 0 °C and then added DMAP (240 mg, 1.97 mmol) and DCC (1218 mg, 5.91 mmol). After 10 h stirring at 25 °C, 4-hydroxy benzaldehyde (2406 mg, 19.7 mmol) was charged and allowed to proceed under stirring for another 36 h at 25 °C. The turbid reaction mixture was filtered to remove the insoluble dicyclohexylurea (DCU), and the filtrate was precipitated in cold hexane. The procedure of precipitation was performed at least 3 times to collect the desired product mPEG-APCL 861 mg (yield: 85%).^1^H NMR (600 MHz, CDCl_3_) δ 9.96 (s, CHO), 7.90 (s, C_6_H_4_), 7.27 (s, C_6_H_4_, overlapped with CDCl_3_), 4.14 (s, OCH_2_), 3.78–3.64 (d, OCH_2_ and CH_2_CH_2_O), 3.55–3.47 (m, OCH), 3.37 (s, CH_3_O), 2.80–2.79 (d, COCH_2_), 2.37–2.35 (t, COCH_2_), 1.87–1.89 (d, CH(CH_2_)_2_).

#### 2.3.2. Synthesis of mPEG-PCL-Imi-DOX

The mPEG-PCL-Imi-DOX was prepared via the condensation of the aldehyde groups of mPEG-APCL and the amino group of DOX. In a typical example, mPEG-APCL (150 mg, equiv 174.8 μmol-CHO) and a catalytic amount of acetic acid were dissolved in anhydrous MeOH and then the solution of DOX·HCl (26 mg, 43.8 μmmol) in DMSO (5 mL) containing an excessive amount of TEA was added dropwise. The Schiff base reaction proceeded at room temperature for 12 h with vigorous stirring. Subsequently, the mixture was concentrated to remove the solvent and then the residue was purified by dialyzing (MWCO = 2000 Da) and freeze-drying. Yield: (114 mg, 73%). ^1^H NMR (600 MHz, DMSO-d6) δ 9.96 (s, CHO), 8.30 (s, CH=N), 7.94–7.75 (s, C_6_H_4_ and the aromatic protons of DOX), 7.32 (s, C_6_H_4_), 5.33 (s, OCH), 5.21 (s, OCH_3_), 4.96 (s, COCH_2_ and phenyl–CH_2_), 4.59–4.57 (s, OCH and HOCH), 4.03 (s, OCH_2_), 3.67–3.50 (d, CH_2_CH_2_O and OCH_2_), 3.24 (s, CH_3_O), 3.12 (s, NCHCH_2_), 2.80 (s, COCH_2_), 2.30 (s, COCH_2_), 2.10 (s, CH_3_), 1.69 (s, CH(CH_2_)_2_).

### 2.4. Structural Characterization

The ^1^H NMR spectra of synthesized polymers were recorded employing a BRUKER AVANCE III-HD 600 MHz spectrometer (Bruker, Karlsruhe, Germany) in CDCl_3_ or DMSO-d_6_. Meanwhile, the DOX prodrug and its precursor were characterized by a Fourier transform infrared spectrometer (BRUKER VERTEX 70V, Bruker, Karlsruhe, Germany). The DOX loading content of the prodrugs was measured using UV-vis spectrophotometry (λ_abs_ = 480 nm, Unico UV-2000, Unico, Dayton, NJ, USA).

### 2.5. Preparation and Characterization of mPEG-PCL-Imi-DOX Micelles

mPEG-PCL-Imi-DOX micelles were prepared by a simple solvent evaporation method. Typically, the DOX prodrug was dissolved in anhydrous THF and stirred for 30 min. The homogeneous solution was added dropwise into deionized water (D.W.) equipped with a magnetic stirring bar and the resulting mixture was stirred at 250 rpm for 24 h. Subsequently, the micellar solution was centrifuged and passed through 0.22 μm microfiltration membrane to remove any solids. Then the collected aqueous phase was lyophilized to obtain the micellar nanoparticles.

The hydrodynamic diameters and zeta potential of resulting micelles were evaluated using dynamic light scattering (DLS, Malvern Zetasizer Nano S90, Malvern Panalytical Ltd, Malvern, UK) in D.W. at 25 °C (*n* = 3); the size and morphology of mPEG-PCL-Imi-DOX micelles at a concentration of 0.5 mg/mL were examined via transmission electron microscopy (TEM, Hitachi HT7700-SS, Hitachi, Tokyo, Japan) with the accelerating voltage of 200 kV.

### 2.6. In Vitro Drug Release

The in vitro DOX release profiles of mPEG-PCL-Imi-DOX micelles were investigated at 37 °C in phosphate buffer solutions (PBS) with different pH values (i.e., 5.0, 6.5 and 7.4). Typically, 5 mL micelles solution (1 mg/mL) in a dialysis bag was immersed and suspended in 30 mL PBS of corresponding pH in darkness with constant stirring at 100 rpm. At designated time intervals, 3 mL of medium solution was aspirated and charged with an equal volume of fresh PBS. The amount of DOX released from mPEG-PCL-Imi-DOX micelles was determined using UV-vis spectrophotometry (λ_abs_ = 480 nm, Unico UV-2000).

### 2.7. In Vitro Cellular Uptake

The cellular uptake of mPEG-PCL-Imi-DOX micelles was examined utilizing MCF-7 cells. Owing to the red fluorescence exhibited by DOX, the study of intracellular uptake could be performed directly without employing any fluorescent probe. Briefly, MCF-7 cells were seeded into a 24-well plate with a density of 5 × 10^3^ cells/well, and incubated with DMEM medium at 37 °C under a humidified atmosphere with 5% CO_2_. After 24 h for incubation, the culture medium was removed and replaced with DMEM containing mPEG-PCL-Imi-DOX micelles for 1, 2 and 4 h. The mixture in each well was discarded, and then the cells were washed with PBS three times and fixed using 100% ethanol for 10 min at ambient temperature. The cell nuclei were counterstained using Hoechst 33342. The corresponding fluorescence microimages were acquired by a confocal laser scanning microscope (CLSM, Nikon C3, Nikon Corporation, Tokyo, Japan).

### 2.8. In Vitro Cytotoxicity Assay

The in vitro cytotoxicity of the micellar nanoparticles prepared with mPEG-APCL and mPEG-PCL-Imi-DOX was assessed by a standard MTT assay with MCF-7 cell line. Briefly, MCF-7 cells (5 × 10^3^ cells per well) were seeded in 96-well plates and incubated for 24 h under an atmosphere with 5% CO_2_ at 37 °C. The cells were treated with various concentrations of the micellar nanoparticles and cultured for another 48 h. The medium was discarded and replaced by 100 μL DMSO, and the absorbance at 490 nm was recorded on a microplate reader.

## 3. Results and Discussion

### 3.1. Polymer Synthesis and Characterization

**Figure 1:**
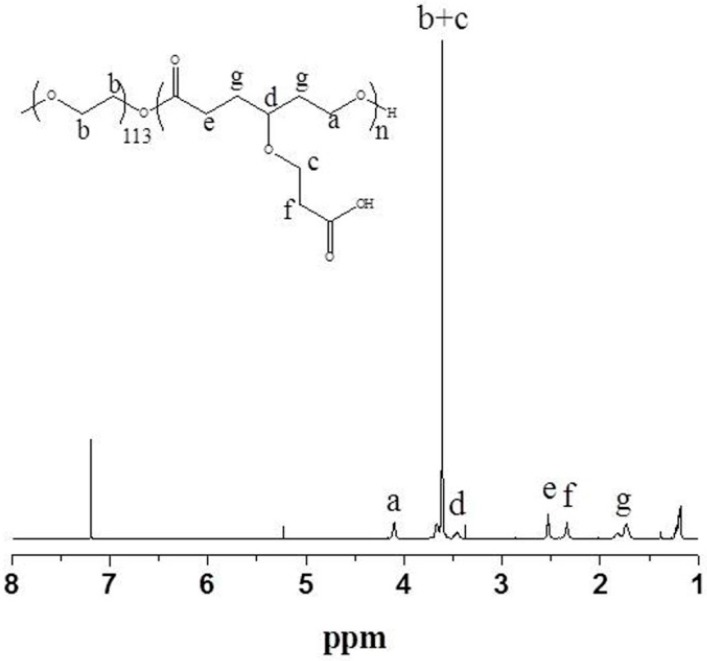
^1^H NMR spectrum of mPEG-CPCL.

**Figure 2:**
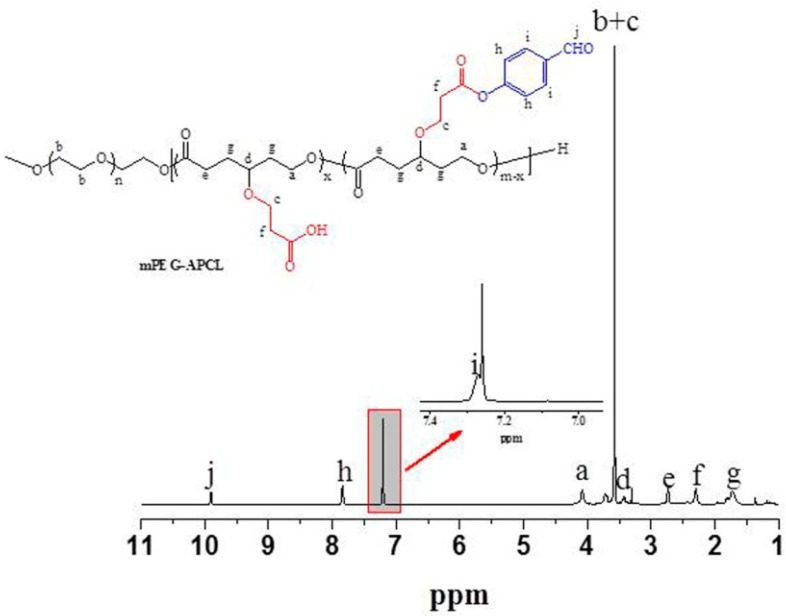
^1^H NMR spectrum of mPEG-APCL.

**Scheme 1:**
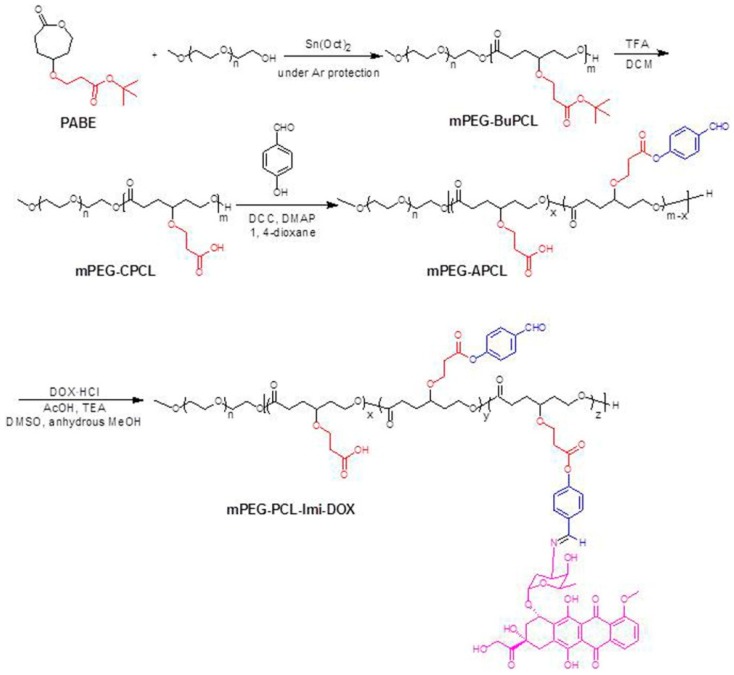
Synthetic route of imine-linked doxorubicin (DOX) prodrugs based on PEG-PCL di-block copolymer.

The primary amino group of DOX condensed with the functional reactive aldehyde groups of mPEG-PCL diblock polymer to form the pH-sensitive imine bonds (Scheme 1). mPEG-BuPCL block copolymer was acquired by controlled ROP of PABE using mPEG5000 as a macroinitiator and Sn(Oct)_2_ as a catalyst. According to the ^1^H NMR spectra of mPEG-BuPCL, it clearly illustrated characteristic hydrogen signals of PEG (δ 3.63 and 3.37 ppm) and BuPCL (δ 4.15–4.11, 3.65–3.63, 3.45–3.41, 2.44–2.42, 2.38–2.34, 1.89–1.72, and 1.44 ppm) (Figure S1, Supplementary Materials). The degree of polymerization for BuPCL block was calculated to be approximately 20 by comparing relative integrals of peaks at δ 4.15–4.11 and 3.63 ppm. Subsequently, after acidolysis deprotection employing excessive trifluoroacetic acid, the corresponding mPEG-CPCL with functional carboxylic groups was obtained. The thorough hydrolysis of all the *tert*-butyl esters was confirmed due to the disappearance of the peak attributed to the *tert*-butyl groups at δ 1.44 ppm (Figure 1). The introduction of aldehyde groups was achieved via the esterification between 4-hydroxy benzaldehyde and mPEG-CPCL employing DCC and DMAP. The newly appeared signals at δ 9.96, 7.90, and 7.27 ppm demonstrated the successful synthesis of mPEG-APCL containing pendant aldehyde groups. The number of benzaldehyde moieties conjugated with each block polymer chain was estimated to be around 12 according to ^1^H NMR integration using signals at δ 7.90 ppm (two aromatic protons of benzaldehyde) versus δ 3.64 ppm (Figure 2).

**Figure 3:**
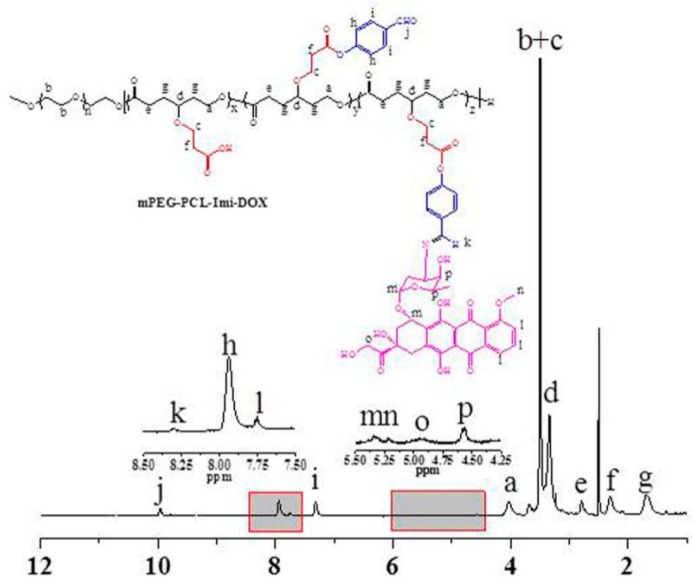
^1^H NMR spectrum of mPEG-PCL-Imi-DOX.

**Figure 4:**
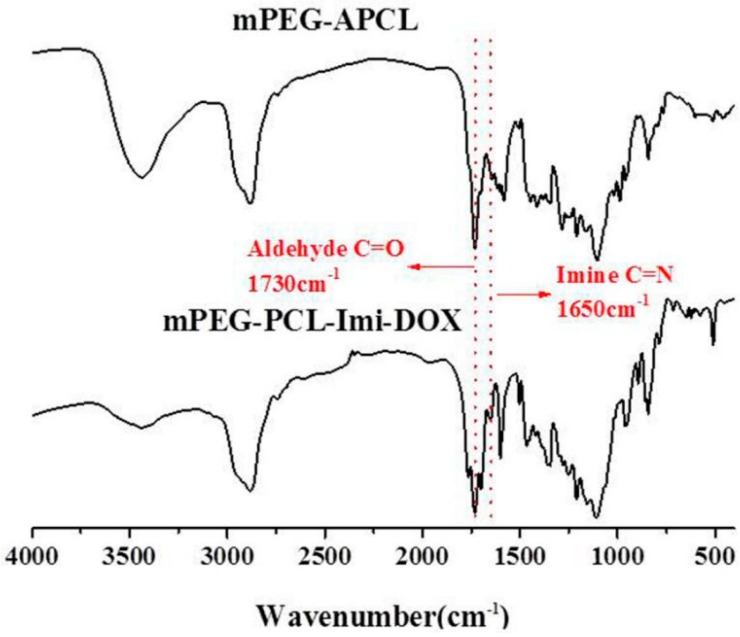
FT-IR spectra of mPEG-APCL and mPEG-PCL-Imi-DOX (24.2 wt % DOX content).

Finally, DOX was readily condensed with aldehyde functionalized mPEG-APCL through a Schiff base reaction to fabricate the pH-sensitive DOX prodrugs. The ^1^H-NMR spectrum of DOX polymeric prodrug (Figure 3) displayed besides the corresponding peaks assigned to the methine protons of CH=N at δ 8.30 ppm also signals due to DOX at 7.94–7.75, 5.33, 5.21, 4.96, 4.59–4.57, 3.12, and 2.10 ppm. For further confirmation of the covalent conjugation between DOX and mPEG-APCL, the FT-IR spectra of mPEG-PCL-Imi-DOX and its precursor were measured. As shown in Figure 4, the new signal at 1650 cm^−1^ assigned to the stretching vibration of C=N [22] demonstrated the formation of the Schiff base linkage. DOX loading content was determined using UV-vis spectrophotometry. The results showed that the DOX content was 13.7, 24.2, and 32.4 wt %, which were obtained by adjusting DOX to mPEG-APCL molar ratios. It is worthwhile to note that these DOX prodrugs possess relatively higher drug loading contents compared with the previously reported PEG-DOX/Ce6 NP (17.95–18.53 wt % DOX) [30] and DOX-PDMA-PBA (11.5–16.2 wt % DOX) [23].

### 3.2. Characterization of mPEG-PCL-Imi-DOX Micelles

**Figure 5:**
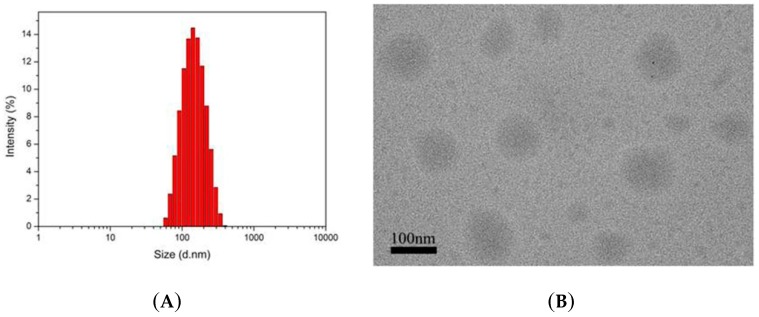
Size distribution profiles of mPEG-PCL-Imi-DOX Micelles determined by DLS (**A**) and TEM (**B**).

DOX prodrugs self-assembled into nano-sized micelles via the simple solvent evaporation method. Based on the comprehensive consideration of solubility and DOX loading content, the size distribution and morphology of mPEG-PCL-Imi-DOX (with 24.2 wt % DOX content) micelles were investigated employing DLS and TEM. The DLS measurement result (Figure 5A) indicated the unimodal size distribution of the mPEG-PCL-Imi-DOX micelles with the mean particle size of 125 nm and a low polydispersity (PDI) of 0.20. The zeta potential analysis of prodrug micelles (Figure S2, Supplementary Materials) displayed a negative surface charge (−10.8 mV), which will offer the improved blood compatibility and prolonged circulation time of nanoparticles due to the reduced interactions with blood constituents [31]. TEM micrograph (Figure 5B) showed that the well-dispersed mPEG-PCL-Imi-DOX micelles formed the spherical morphology possessing an average diameter of ca. 110 nm, somewhat smaller than that measured by DLS, most likely because of the shrinkage of the micelles upon drying.

### 3.3. In Vitro Drug Release of mPEG-PCL-Imi-DOX Micelles

**Figure 6:**
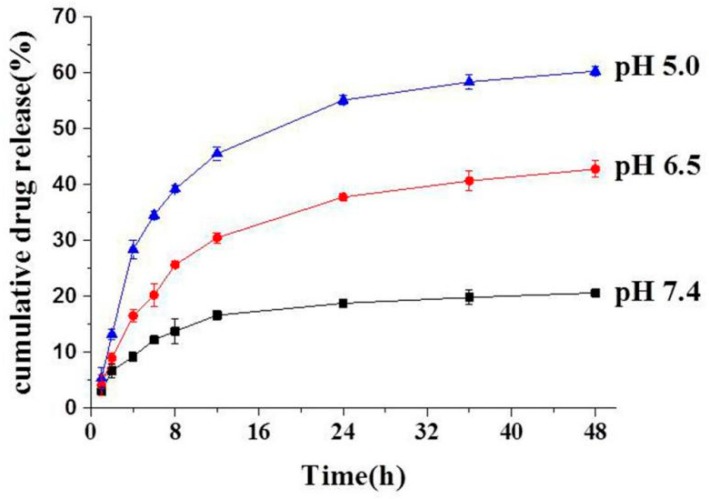
DOX release profiles of mPEG-PCL-Imi-DOX micelles under different pH levels. Data were presented as mean ± standard deviation (SD) (*n* = 3).

The in vitro drug release was determined utilizing PBS of three different pH levels, mimicking the acidic microenvironment of extra- and intra-tumor cells (pH 5.0 and 6.5) and the physiological condition of normal tissues (pH 7.4). As depicted in Figure 6, about 20% of the covalently conjugated DOX was released from mPEG-PCL-Imi-DOX micelles at pH 7.4 for 48 h. However, the cumulative drug release from prodrug micelles increased up to 42.8 ± 1.5% and 60.3 ± 0.8% at pH 6.5 and 5.0 after 48 h, respectively. These data demonstrated that the DOX released from prodrug micelles could be accelerated along with the decrease of pH values, which is likely ascribed to the destruction of imine bonds. Meanwhile, no burst release was observed at all three different pH conditions. All the results indicated that the designed prodrug micelles offered an impactful approach for the sustainable release of antitumor drugs under acidic microenvironment of tumors.

### 3.4. In Vitro Cellular Uptake and Cytotoxicity Studies of mPEG-PCL-Imi-DOX Micelles

**Figure 7:**
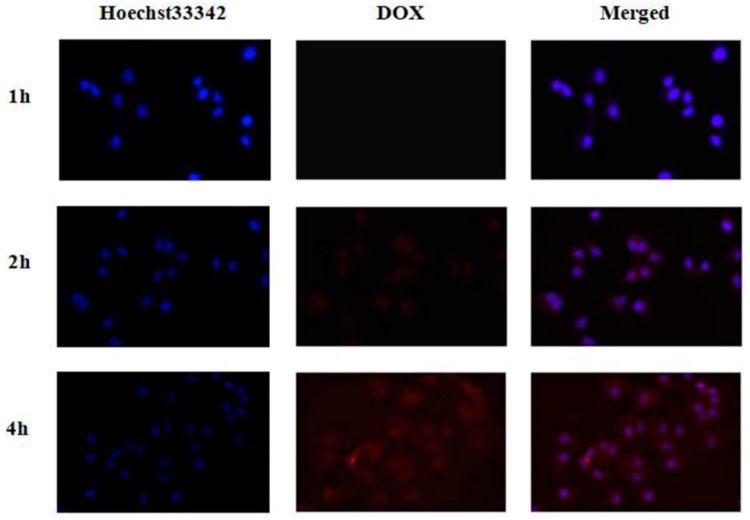
Confocal laser scanning microscopy images of MCF-7 cells treated with DOX prodrug micelles for 1, 2 and 4 h.

The cellular uptake test of mPEG-PCL-Imi-DOX micelles was evaluated in MCF-7 cells utilizing confocal laser scanning microscopy. Thanks to the intrinsic fluorescent properties of DOX, DOX prodrug micelles showed red fluorescence, while the cell nuclei stained by Hoechst 33342 exhibited blue fluorescence. MCF-7 cells were incubated with micelles conjugated with DOX for 1, 2, and 4 h, respectively. As illustrated in Figure 7, along with the prolongation of incubation time, the red fluorescence intensity in MCF-7 cells cytoplasm increased tremendously. The phenomenon suggested that DOX prodrug micelles were efficiently internalized by the cells.

**Figure 8:**
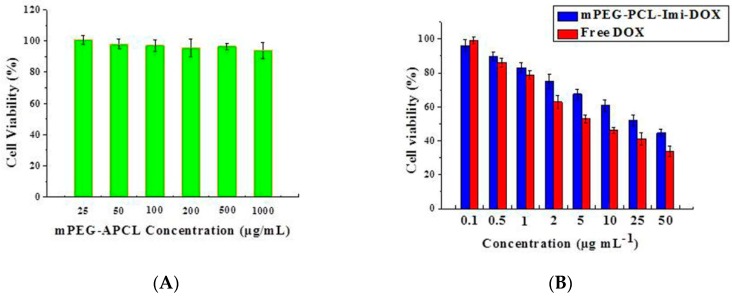
Cell viabilities of MCF-7 cells co-incubated with mPEG-APCL (**A**) and free DOX and mPEG-PCL-Imi-DOX (**B**). Data were presented as mean ± standard deviation (SD) (*n* = 3).

Biocompatibility of the drug carrier is the vital factor, which should be considered in the field of DDS. Therefore, we first investigated the cytocompatibility of the blank micelles fabricated with mPEG-APCL via the MTT assay. Figure 8A displayed that the blank micelles exhibited no obvious cytotoxicity to MCF-7 cells. The cell viability was still more than 90% even with the concentration up to 1000 μg/mL. This result indicates that mPEG-APCL is of extremely low cytotoxicity and possesses the excellent biocompatibility.

Subsequently, the in vitro cell proliferation inhibition efficacies of free DOX and mPEG-PCL-Imi-DOX nano-object against MCF-7 cells were evaluated. As illustrated in Figure 8B, all the samples exhibited a dose-dependent cytotoxicity after 48 h co-culture for 48 h. The result of the biocompatibility test showed that mPEG-APCL exhibited negligible cytotoxicity towards MCF-7 cells. Hence, it is reasonable to deduce that the in vitro antitumor effect of mPEG-PCL-Imi-DOX nanoparticles is attributed to the conjugated DOX. Compared with free DOX, the inhibitory effect of mPEG-PCL-Imi-DOX micelles was slightly weaker (IC_50_ 26.42 vs. 9.58 μg·mL^−1^). This phenomenon could be explained by the rapid diffusion of free DOX and slow release of DOX from mPEG-PCL-Imi-DOX. This result demonstrates that the formulation of DOX prodrug is able to reduce the cytotoxicity against MCF-7 cells leading to the reduced side effects of DOX.

## 4. Conclusions

In summary, a novel pH-responsive polymeric prodrug based on PEG-PCL has been synthesized and employed for the delivery of DOX. The amphiphilic DOX prodrug with relatively higher drug loading content can easily self-assemble into nano-sized micelles, which exhibit a unimodal size distribution. The in vitro drug release test reveals that DOX release from the prodrug micelles is of a pH-dependent manner and can be accelerated upon exposure to the moderately acidic microenvironment of tumors. Meanwhile, the results of in vitro cellular uptake and cytotoxicity studies indicate that these prodrug micelles are able to be efficiently taken up and alleviate the side effects of DOX. Taken together, the developed DOX prodrug micelles with imine bonds are promising alternatives for the targeted release of anticancer therapeutics.

## Supplementary Materials

The following are available online at http://www.mdpi.com/2073-4360/10/10/1127/s1, Figure S1: ^1^H NMR spectrum of mPEG-BuPCL Block Copolymer, Figure S2: Zeta potential of mPEG-PCL-Imi-DOX Micelles.
