# Supplementary material for: Design, Synthesis, and Characterization of Schiff Base Bond-Linked pH-Responsive Doxorubicin Prodrug Based on Functionalized mPEG-PCL for Targeted Cancer Therapy
Source: Polymers (Basel). 2018 Oct 11;10(10):1127. doi: 10.3390/polym10101127 (PMC6404085; doi:10.3390/polym10101127)
Supplement: Supplementary file 1 [file polymers-10-01127-s001.pdf]

Supplementary Materials

Design, Synthesis, and Characterization of Schiff Base Bond-Linked pH-Responsive Doxorubicin Prodrug Based on Functionalized mPEG-PCL for Targeted Cancer Therapy

Yinglei Zhai [1,†], Xing Zhou [2,†], Zhiqiang Zhang [3], Lei Zhang [4], Dianyu Wang [1], Xinhui Wang [1], Wei Sun [1,*]

[1] Department of Biomedical Engineering, School of Medical Devices, Shenyang Pharmaceutical University, Shenyang 110016, China; yingleizhai@syphu.edu.cn (Y.Z.); wdy545820@163.com (D.W.); m15141210344@163.com (X.W.)

[2] Hainan Institute of Materia Medica, Haikou 570311, China; beyondyme@163.com

[3] Department of Pharmaceutics, School of Pharmacy, Shenyang Pharmaceutical University, Shenyang 110016, China; zifeiyuxq@163.com

[4] Shanghai Pharma Group (Benxi) Northern Pharmaceutical Co., Ltd, Benxi 117004, China; zl.719@163.com

* Correspondence: sunwei@syphu.edu.cn or sunwei19801208@163.com

† These two authors contributed equally to this work.

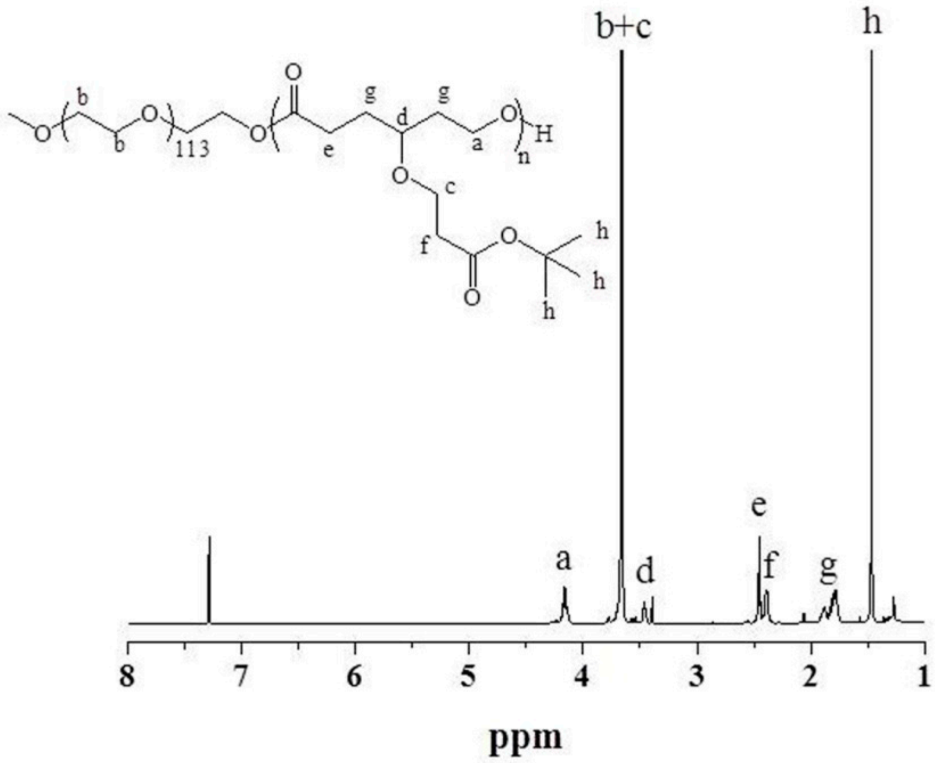

Figure S1. ^{1}H NMR spectrum of mPEG-BuPCL Block Copolymer.

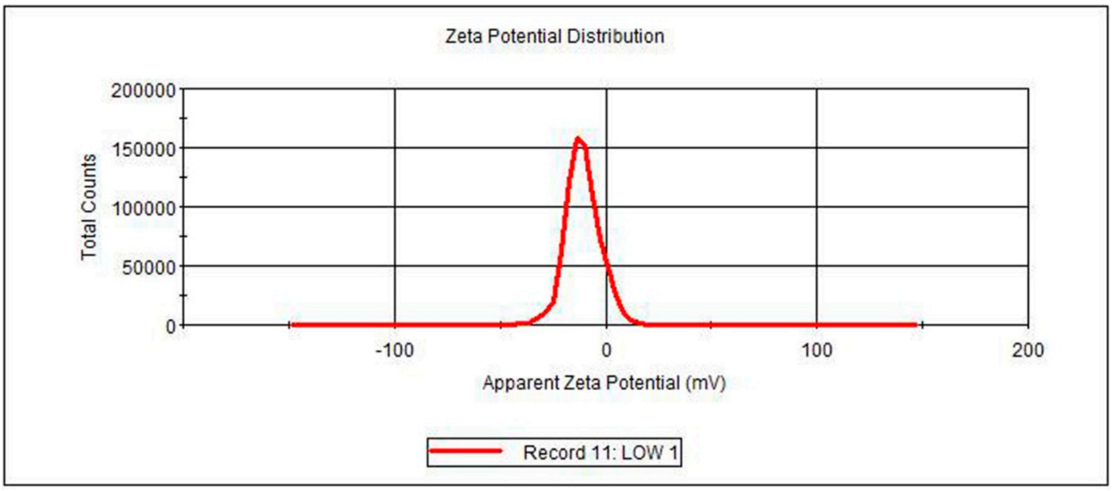

Figure S2. Zeta potential of mPEG-PCL-Imi-DOX Micelles.
